# Tipificación bioquímica y evaluación de la patogenicidad de aislamientos vulvovaginales del complejo *Candida albicans*

**DOI:** 10.7705/biomedica.6861

**Published:** 2023-08-31

**Authors:** Soraya Morales-López, Keiner Ustate, Zulay Pedrozo, Yulibeth Torres

**Affiliations:** 1 Grupo CINBIOS, Programa de Microbiología, Universidad Popular del Cesar, Valledupar, Colombia Universidad Popular del Cesar Grupo CINBIOS Universidad Popular del Cesar Valledupar Colombia; 2 Laboratorios Nancy Flórez García S.A.S., Valledupar, Colombia Laboratorios Nancy Flórez García S.A.S Valledupar Colombia

**Keywords:** Candida albicans, candidiasis, biopelículas, Candida albicans, candidiasis, biofilms

## Abstract

**Introducción.:**

*Candida albicans, C. dubliniensis* y *C. africana* forman el complejo *Candida albicans.*

**Objetivo.:**

Identificar las características fenotípicas y patogénicas de aislamientos del complejo *C. albicans* conservados en una colección.

**Materiales y métodos.:**

Se evaluaron 300 aislamientos identificados presuntivamente como del complejo *C. albicans,* utilizando CHROMagar^TM^ *Candida.* Se determinó la producción del tubo germinal mediante tres métodos, se evaluó la producción de clamidosporas, se caracterizaron las colonias en agares artesanales *(Rosmarinus officinalis* y *Nicotiana tabacum)* y se utilizó MALDI-TOF como prueba de referencia para la identificación. Para detectar factores de patogenicidad, se evaluó la actividad hemolítica de los aislamientos independientes y en cocultivo con *Staphylococcus aureus,* la producción de enzima coagulasa y la formación de biopelículas.

**Resultados.:**

El 43,7 % de los aislamientos produjo tubo germinal en caldo de medio infusión de cerebro-corazón y el 47 % generó clamidosporas. En los medios artesanales, en el 6 % de los aislamientos se obtuvieron colonias de color café en agar romero y, en el 5 %, en agar tabaco. Ninguna de las cepas hemolizó el agar sangre comercial (ni en presencia o ausencia de *S. aureus),* mientras que el 50 % hemolizó el agar papa dextrosa suplementado con sangre. Todos los aislamientos produjeron enzima coagulasa y la producción de biopelículas fue variable. Para la producción de tubo germinal, el método de suero humano mostró igual positividad que el de caldo de leche. Todos los aislamientos fueron identificados como *C. albicans* por MALDITOF.

**Conclusiones.:**

Se requieren herramientas de proteómica y pruebas moleculares, o la combinación de métodos, para poder discriminar entre especies.

## Abstract (en)

**Introduction.:**

*Candida albicans, C. dubliniensis,* and *C. africana* form the *Candida albicans* complex.

**Objective.:**

To identify the phenotypic and pathogenic characteristics of isolates of the *C. albicans* complex preserved in a collection.

**Materials and methods.:**

Three hundred presumptive strains of the *C. albicans* complex were evaluated using CHROMagar^TM^ *Candida.* Germ tube production was determined by three methods, chlamydospores formation was assessed and colonies were characterized in artisanal agars *(Rosmarinus officinalis* and *Nicotiana tabacum).* MALDI-TOF was used as the gold standard identification test. To detect pathogenicity factors, we evaluated the hemolytic activity of each isolate and cocultured with *Staphylococcus aureus,* coagulase enzyme production, and biofilm formation.

**Results.:**

Out of the 300 isolates, 43.7% produced germ tube in the heart-brain infusion broth and 47% of the isolates produced chlamydospores. In the artisan media, 6% of the isolates produced brown colonies on rosemary agar and 5% did so on tobacco agar. None of the strains hemolyzed the blood agar alone or cocultured with *S. aureus.* However, 50% of the isolates hemolyzed the potato dextrose agar supplemented with blood. All strains were coagulase producers, and biofilm production was variable. For germ tube production, the human serum method showed the same positivity as the milk broth method. All isolates were identified as *C. albicans* by MALDI-TOF.

**Conclusions.:**

The use of proteomics, molecular tests or a combination of methods is required for species identification.

*Candida albicans* es un complejo de especies crípticas o estrechamente relacionadas que incluye a *C. albicans sensu stricto, C. dubliniensis* y *C. africana.* En la actualidad, *C. albicans sensu stricto* es reconocida como el agente causal más común en las infecciones micóticas ^1^^,^^2^.

*Candida dubliniensis* y *C. africana* se consideraron inicialmente como especies "menores" o variedades biológicas de *C. albicans,* y se reportaron como "cándidas atípicas" por compartir características fenotípicas. Hoy, *C. dubliniensis* se reconoce como una especie y *C. africana* se considera una variedad de *C. albicans*^3^.

Entre las características estudiadas mediante pruebas fisiológicas para diferenciar las especies del complejo, se encuentran el color de las colonias en los medios cromógenos, el crecimiento a 45 °C, la producción de clamidosporas, la morfología de las colonias en agar Staib o agar tabaco y la inhibición del crecimiento en medio con concentraciones altas de sal, etc. No obstante, ninguna prueba es completamente específica ^4^^,^^5^.

*Candida dubliniensis* fue descrita por primera vez en 1995 6 y se reconoce como una levadura capaz de producir clamidosporas y tubos germinativos, al igual que *C. albicans. C. dubliniensis* es un agente patógeno significativamente menos virulento y versátil, que se ha recuperado en ubicaciones geográficas en diversas zonas geográficas ^7^. En Colombia, este microorganismo fue identificado por primera vez mediante métodos fenotípicos en 2008 ^8^.

*Candida africana es* la especie emergente más reciente de este complejo, posee tubo germinal, no asimila N-acetilglucosamina y no produce clamidosporas. Se aisló por primera vez a partir de muestras de pacientes de África y Alemania ^9^ y, desde el 2001, se considera una variedad de *C. albicans (C. albicans* var. *africana)*^3^^,^^10^. Se ha identificado en países como Chile, Nigeria, Irán y Argentina ^1^^,^^3^^-^^4^^,^^11^^,^^12^. En Colombia, la identificación molecular de *C. africana* a partir de cepas caracterizadas previamente como *C. albicans* atípicas, arrojó resultados sugestivos de *C. albicans* ^*(*^^5^. En la actualidad, la identificación definitiva de *C. africana* requiere pruebas adicionales, como técnicas moleculares para detectar el gen *hwp1*^13^^,^^14^.

Por otra parte, y en relación con los factores determinantes de la patogénesis (producción de hemolisinas, biopelículas y enzimas), con los recientes estudios se demuestra el creciente interés en aislamientos de *C. albicans* y *C. dubliniensis*^15^^-^^17^. Se sabe que todos estos factores patogénicos juegan un rol importante en el proceso infeccioso, la colonización y la permanencia del hongo en la superficie corporal, además de la formación de poros y el efecto lítico en las células eucarióticas; no obstante, aún es mucho lo que se desconoce.

El objetivo de este trabajo fue identificar las características fenotípicas y patogénicas de aislamientos vulvovaginales del complejo *Candida albicans,* mediante su cultivo en diferentes medios enriquecidos, la evaluación de la actividad de las hemolisinas y la formación de biopelículas.

## Materiales y métodos

Se estudiaron 300 aislamientos de levaduras, recolectados durante los años 2018 y 2019, y conservados en una colección de cultivos. Las cepas se aislaron de frotis vulvovaginales de igual número de pacientes adultas, con diagnóstico presuntivo de candidiasis vulvovaginal, quienes asistieron a dos laboratorios clínicos de Valledupar.

Para la recuperación de las cepas, las muestras se sembraron en agar Sabouraud y se incubaron a 37 °C durante 48 horas. Los tubos en los que no se observó crecimiento, se resembraron en caldo infusión de cerebro-corazón *(Brain Heart Infusión,* BHI) con enriquecimiento Fildes.

### Identificación morfológica y fenotípica

*CHROMagar™ Candida.* Las cepas aisladas se sembraron en CHROMagar™ *Candida* y se incubaron a 35 °C por 48 horas. Se seleccionaron las colonias de color verde por considerarse presuntivas del complejo *C. albicans.*

Como controles, se emplearon las cepas de *C. albicans* de la *American Type Culture Collection* (ATCC), con números de catálogo ATCC 10231 y ATCC 90028; *C. africana* MYA-2669 y *C. dubliniensis* - ATCC 3949.

*Formación de tubo germinal.* La formación del tubo germinal se evaluó con caldo de leche, caldo infusión de cerebro-corazón y suero humano. Para todos los casos, se preparó un inóculo (10^5^ a 10^6^ células por ml) con una colonia aislada y joven -menos de 48 horas- y se homogenizó en tubos que contenían 5 ml de caldo de leche o caldo infusión de cerebro-corazón o 0,5 ml de suero humano, y se incubaron a 37 °C. Después de dos horas, se hicieron los montajes entre lámina y laminilla, y se observaron microscópicamente con ayuda de un objetivo que amplifica 400 veces, en busca de prolongaciones filamentosas ^18^.

*Formación de clamidoconidias.* Para la producción de clamidoconidias, se usó el mismo tubo de la prueba para tubo germinal, cuya incubación a 37 °C se extendió hasta por cinco días. Se realizó la lectura por microscopía óptica en busca de estructuras redondas con pared celular gruesa ^4^.

*Crecimiento en agar romero (Rosmarinus officcinalis) y agar tabaco (Nicotiana tabacum).* Para la diferenciación presuntiva de *C. dubliniensis,* cada cepa se sembró por agotamiento en agar romero *(Rosmarinus officinalls)* y agar tabaco *(Nicotiana tabacum),* y se incubó por 4 a 5 días a 37 °C.

En el agar romero, se inspeccionó la apariencia de la colonia y se determinó la presencia o ausencia de clamidoconidias ^19^. Se consideraron aislamientos presuntivos de *C. dubliniensis* aquellos con colonias rugosas, bordes de hifas periféricas y abundantes clamidosporas; mientras que se presumieron como aislamientos de *C. albicans* aquellos con colonias lisas y sin clamidosporas.

En el agar tabaco, se analizaron el color y el aspecto de la colonia, y la formación de clamidoconidias. Se consideraron aislamientos presuntivos de *C. dubliniensis* aquellos con colonias rugosas de color café y con abundantes clamidoconidias ^20^^,^^21^. Se presumió que los aislamientos con colonias lisas blancas o crema y sin clamidoconidias, correspondían a *C. albicans.*

### Evaluación de la patogenicidad

*Actividad hemolítica.* La actividad hemolítica se evaluó en agar sangre de carnero (Ad-bio- ANNAR™) y agar papa dextrosa (Scharlau™) suplementado con 5 % de sangre fresca de carnero y 3 % de glucosa. Por cada aislamiento se marcó una estría sobre el agar y las placas se incubaron a 37°C, por 48 horas, en atmósfera microaerofílica ^22^. Las hemólisis beta (total) y la alfa (parcial) se consideraron resultados positivos. Se emplearon aislamientos de *S. aureus* (ATCC 25923) y *C. albicans* (ATCC 10231) como controles positivos en agar sangre de carnero y agar PDA con sangre, respectivamente.

*Efecto cohemolítico o factor CAMP como indicador de hemólisis de tipo beta en cocultivo con Staphylococcus aureus.* Se evaluó la producción de factor CAMP como indicador de hemólisis de tipo beta en cocultivo con *S. aureus.* Se hizo una siembra perpendicular con una estría de *S. aureus* ATCC 25923, a una distancia de 10 mm desde el borde de la colonia de la levadura. Las placas se incubaron a 37 °C por 48 horas en atmósfera microaerofílica, en búsqueda de una deformación en punta de flecha en la intersección de las hemólisis ^15^^,^^17^^,^^23^. Como controles positivos, se utilizaron las cepas de referencia *S. aureus* (ATCC 25923) y *S. agalactiae* (ATCC 12386).

*Formación de biopelículas.* Para la formación de biopelículas, se prepararon soluciones en tubos de poliestireno cónico, con tapa de rosca, con 5 ml de caldo Sabouraud dextrosa al 8 % inoculado con una colonia de cada aislamiento (10^5^ a 10^6^ células por ml). Los tubos se incubaron a 35 °C en el equipo *Shaking Incubator* (Being^TM^ THZ-300) con agitación constante a 75 rpm por 72 horas. Se aspiró el contenido del caldo y se hicieron dos lavados con agua destilada. Para evidenciar la formación de la biopelícula, se agregaron 3 ml de safranina al 1, 5 y 10 % por 10 minutos y se decantó por inversión. Los resultados se interpretaron como positivo (fuerte, moderado, débil) o negativo (ausente), por inspección visual de una capa de biopelícula adherente en la parte inferior y en la pared de los tubos ^15^^,^^17^. Se emplearon *C. albicans* (ATCC 10231) como control positivo y solución salina fisiológica como control negativo.

*Producción de coagulasa.* A partir de un subcultivo en agar Sabouraud (incubado a 37 °C durante 48 horas), se inoculó una colonia de los microorganismos en un tubo con 3 ml de plasma de conejo con EDTA (10^5^ a 10^6^ células por ml). Este se incubó por 4 horas a 37 °C. Los tubos se inclinaron cada media hora de forma suave. La prueba de coagulasa se consideró positiva cuando se observó la formación de un coágulo ^24^. Se usó S. *aureus* (ATCC 25923) como control positivo.

Todas las pruebas se practicaron por triplicado en tres días diferentes.

### Prueba de referencia

Todas las cepas fueron identificadas por espectometría de masas MALDI-TOF (espectometría de masas de tipo desorción-ionización láser, asistida por matriz, acoplada a un detector iónico de tiempo de vuelo) en el equipo Vitek MS™ (bioMérieux), de acuerdo con el protocolo sugerido por el fabricante. La identificación se consideró confiable con un puntaje superior al 98 % ^25^.

## Resultados

### Identificación morfológica y fenotípica

**Figura 1:**
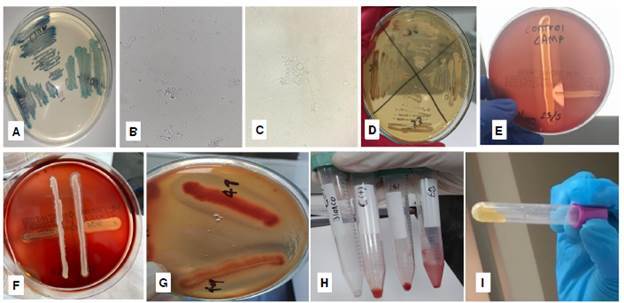
a. Tonalidades de verde en CHROMagar™ *Candida,* b. Imagen de tubos germinales, 400X. c. Producción de clamidoconidias, 400X. d. Colonia café (izquierda) en agar romero. e. Control positivo de la prueba de Christie, Atkins y Munch-Peterson (CAMP) en medio agar con sangre comercial. f. Prueba CAMP negativa en dos aislamientos de *Candida* (líneas verticales). g. Hemólisis generada por dos aislamientos de *Candida* en agar papa dextrosa y sangre, con suplemento de glucosa. h. Controles (negativo y positivo) y resultados positivos de formación de biopelículas (débil/fuerte), con 10% de safranina, en dos aislamientos. i. Prueba positiva para coagulasa.

En el CHROMagar^TM^ *Candida* se observaron colonias en diferentes tonalidades de color verde (figura 1a), sin que fuera posible hacer una clara discriminación que permitiera su cuantificación.

Todos los 300 aislamientos formaron tubo germinal en medio de suero humano y caldo de leche; mientras que 131 (43,7 %) lo hicieron por el método con caldo infusión de cerebro-corazón (figura 1b y cuadro 1). Se obtuvo producción de clamidoconidias en 141 aislamientos (47 %), luego de cinco días de incubación a 37 °C (figura 1c y cuadro 1).

**Cuadro 1:** Características fenotípicas y determinantes de la patogenicidad evaluadas en los aislamientos del complejo *Candida albicans.* Los valores están expresados en porcentaje.

Tubo germinal			CLAM		Agar romero		Agar tabaco			Hemólisis y CAMP en agar sangre	Hemólisis y CAMP en agar papa dextrosa	Producción de biopelículas			Coag.	Identificación por MALDI-TOF
					Aspecto por MALDI-TOF de la colonia		CLAM	Color y aspecto de la colonia		CLAM						
Suero	Caldo leche	Caldo infusión de cerebrocorazón	Pos	Neg	Rugosa	Pos	Café/ rugoso	Blanco/ liso	Pos	Pos	Pos	Débil	Moderado	Fuerte	Pos	*Candida* *albicans*
100	100	43,7	47	53	0	0	5	95	0	0	50	24,8	28,7	28,1	100	100

CLAM: clamidoconidias; CAMP: prueba de Christie, Atkins y Munch-Peterson para detectar *Streptococcus agalactiae;* AS: agar sangre comercial; PDA: agar papa dextrosa: agar papa dextrosa con suplemento 3 % de glucosa al 3 % y sangre de carnero al 5 %; Coag: prueba de coagulasa; infusión de cerebro-corazón: medio de infusión de cerebro-corazón; Pos: positivo; Neg: negativo; MALDI-TOF: espectometría de masas tipo desorción/ionización láser, asistida por matriz, acoplada a un detector iónico de tiempo de vuelo

En relación con las características de crecimiento en agares artesanales, en el agar romero, se encontró que todas las colonias fueron lisas y sin presencia de hifas periféricas. En el caso del agar tabaco, se encontró que 15 aislamientos (5 %) produjeron colonias de color café; mientras que 285 aislamientos produjeron colonias de color cremablanco. Ningún aislamiento produjo clamidosporas en estos medios (figura 1d y cuadro 1).

### Mecanismos de patogenicidad

La prueba de hemólisis se practicó en agar sangre comercial (Ad-bio^TM^), y agar papa dextrosa (Scharlau^TM^) suplementado con glucosa y sangre de carnero.

Antes de la selección de los medios, se hicieron pruebas con cepas hemolíticas de S. *aureus* (ATCC 25923) y *C. albicans* (ATCC 10231) en agar papa dextrosa suplementado con glucosa y sangre humana, con sangre fresca de carnero y con sangre comercial de carnero, sin que se obtuviera crecimiento del microorganismo bacteriano en ninguno de estos medios.

En agar sangre (Ad-bio™), las colonias de S. *aureus* crecieron y produjeron hemólisis completa, característica de esta cepa (figura 1e). Sin embargo, para este medio, no se encontraron cepas productoras de hemolisinas y con ningún aislamiento se generó hemólisis o punta de flecha en cocultivo con los aislamientos de S. *aureus* (figura 1f y cuadro 1).

En todas las diferentes versiones del agar papa dextrosa con sangre y glucosa, hubo crecimiento y hemólisis del microorganismo *C. albicans* (ATCC 10231). Además, el 50 % de los aislamientos generó hemólisis completa y produjo el factor CAMP (punta de flecha) con el aislamiento de S. *aureus* (figura 1g).

En relación con la producción de biopelículas, los resultados se apreciaron mejor con 10 % de safranina. De los 300 asilamientos, 246 (82 %) fueron positivos para la formación de biopelículas: 85 aislamientos (28 %) lo hicieron de forma fuerte; 86 (29 %), de forma moderada y 74 (25 %) produjeron una biopelícula débil (figura 1h) (cuadro 1). Todos los aislamientos (300) coagularon el plasma durante las dos primeras horas de incubación (figura 1i y cuadro 1).

Espectometría de masas MALDI-TOF. Todos los aislamientos se identificaron como *C. albicans* mediante la técnica de MALDI-TOF, utilizando el equipo Vitek MS.

## Discusión

Las técnicas de secuenciación de ADN han generado avances en el conocimiento de los microorganismos, lo que ha ocasionado cambios sustanciales en la taxonomía de los hongos. Así, las que antes eran consideradas simples morfoespecies han sido sustituidas por complejos de especies crípticas que difieren en virulencia y en su reacción frente a los antifúngicos, de ahí la importancia de su correcta identificación ^26^.

En esta investigación, se encontraron aislamientos con distintas tonalidades de color verde en CHROMagar™ *Candida.* Estos resultados coinciden con los descritos por Vieille *et al.* en 2022, quien empleó este medio para levaduras del complejo *C. albicans* y encontró que sus aislamientos mostraban tonalidades verdes con leves diferencias ^4^.

Igualmente, Ballesté *et al.* (2005), Alfonso *et al.* (2010), Odds *et al.* (2000), Madhavan *et al.* (2011) y Vecchione *et al.* (2017), ya habían descrito antes las levaduras de color verde dentro del complejo *C. albicans*^27^^-^^31^.

En 1998, Sullivan *et al.* describieron las diferencias entre especies utilizando agar CHROMagar™ *Candida: C. albicans* produjo colonias de color azul verdoso claro, mientras que *C. dubliniensis* produjo colonias de color verde oscuro después de 48 horas de crecimiento a 37 °C ^32^. Estos hallazgos también los describieron Romeo y Criseo, quienes encontraron que *C. africana* producía colonias con un tono levemente verde ^13^.

En el presente trabajo, la tonalidad de la colonia no estuvo relacionada con la identificación a nivel de especie. Los hallazgos reportados por otros autores podrían estar relacionados con la preparación del lote del medio, el tiempo de conservación de los aislamientos, etc. ^31^. Cabe mencionar que tampoco fue posible la diferenciación por tonalidad de las cepas *C. albicans, C. africana* y *C. dubliniensis,* empleadas como control.

Por otro lado, la prueba del tubo germinal, conocida también como la de filamentación en suero o filamentación precoz, se consideraba la más sencilla, rápida y económica para la identificación preliminar de los aislamientos del complejo *C. albicans*^18^. En el presente trabajo, los resultados de esta prueba variaron según la técnica empleada: los mayores índices de positividad se obtuvieron con suero humano y con caldo de leche. Estos hallazgos son similares a los descritos en 2008 por Pineda *et al.,* quienes obtuvieron una positividad del 93,8 % ^21^. No obstante, otros estudios, como los de Yazdanpanah y Khaithir (2014), muestran una positividad menor, con variaciones entre el 51 y el 63 % ^33^.

Los aislamientos de *C. albicans* no productores de clamidoconidias pueden considerarse infrecuentes y reconocerse como atípicos. En Colombia, Rodríguez-Leguizamón *et al.* trabajaron con 11 cepas identificadas como *C. albicans* incapaces de producir clamidosporas y cuyas espectrometrías MALDI-TOF las caracterizaron como *C. albicans/C. africana, C. albicans o C. africana.* No obstante, todos los aislamientos fueron tipificados molecularmente como *C. albicans* cuando se amplificó el gen *hwp1*^5^.

En relación con los medios que se emplean para la formación de clamidoconidias, suelen ser variados e incluyen el caldo con leche diluida ^34^^-^^36^. Aunque la temperatura de incubación para el desarrollo de clamidoconidias suele ser entre 28 y 30 °C, algunos autores han realizado incubaciones hasta los 41 °C ^37^. Así, el bajo porcentaje de resultados positivos para la formación de clamidoconidias en esta investigación, podría estar relacionado aislamientos.

A propósito del uso del agar tabaco como medio de cultivo para la discriminación rápida entre *C. dubliniensis* y *C. albicans,* Pineda *et al.* (2008) encontraron que el 100 % de los aislamientos de *C. dubiniensis* produjeron colonias cafés y rugosas, mientras que el 98,5 % de las colonias de *C. albicans* fueron blancas y lisas ^21^. A pesar de que la literatura científica indica que las colonias de color marrón amarillento con bordes festoneados son típicas de *C. dubliniensis*^20^, nuestros resultados arrojaron 15 aislamientos de color café que fueron identificados como *C. albicans* por MALDI-TOF. Es importante recordar que ninguno de esos aislamientos había producido clamidoconidias, por lo que, ante la ausencia de estas estructuras, la similitud con *C. dubliniensis* se limitaba al color de la colonia.

Silveira *et al.* (2011) también obtuvieron una coloración diferente a la esperada con el agar tabaco: los autores describieron las colonias de *C. dubliniensis* como de *C. albicans,* ya que estas presentaron morfotipo y micromorfología típicos de esta última especie ^38^.

Asimismo, el agar romero fue descrito por De Loreto *et al.* (2008) para diferenciar entre *C. dubliniensis* y *C. albicans*^19^ y, aunque es escasa la información bibliográfica que documenta su empleo, en esta investigación se encontró que ninguno de los aislamientos produjo colonias rugosas o con clamidoconidias. Curiosamente, 18 aislamientos (6 %) produjeron colonias de color café, pero de contorno liso y sin clamidoconidias, por lo cual se descartó la presencia de aislamientos típicos de *C. dubliniensis.*

En cuanto a la expresión de mecanismos de patogenicidad, de los 300 aislamientos probados, ninguno fue capaz de producir hemólisis o factor CAMP en el agar sangre preparado y distribuido de forma comercial. Estos resultados son opuestos a los descritos por Pakshir *et al.,* quienes encontraron que todos sus aislamientos (110) produjeron hemólisis completa y casi la mitad de los aislamientos fueron positivos para el factor CAMP con cepas de referencia de S. *aureus* (43,6 %) y S. *agalactiae* (49 %) como controles positivos ^15^.

Por otra parte, en agar papa dextrosa con suplemento de glucosa y sangre de carnero, la mitad de los aislamientos produjo hemólisis completa. Los patrones de hemólisis en las levaduras de este complejo ya se habían descrito anteriormente, con amplia variabilidad en los resultados: Sardi *et al.* (Brasil), Luo *et al.* (China) y, recientemente, Nouraei *et al.* (Irán), encontraron que todos sus aislamientos produjeron hemólisis completa ^39^^-^^41^. Wan *et al.* (China) informaron que todas sus cepas produjeron hemólisis (sin especificar el tipo de hemólisis), mientras que El Baz *et al.* (Egipto) y Noori, *et al.* (Irán), reportaron que la hemólisis fue completa solo en el 79 y el 28 % de los aislamientos estudiados, respectivamente ^42^^-^^44^. Estas variaciones en el patrón de hemólisis podrían estar asociadas con los genotipos circulantes en cada región geográfica.

Todos estos autores emplearon agar Sabouraud dextrosa con 5-7 % de sangre de carnero fresca y 3 % de glucosa. En otro estudio, Maheronnaghsh *et al.* emplearon sangre humana y obtuvieron hemólisis en más del 70 % de los aislamientos ^45^.

La ausencia de hemólisis fue descrita en 1994 por Manns *et al.* al emplear placas de agar Sabouraud o tripticasa de soya con sangre, pero sin enriquecimiento con 3 % de glucosa, o cuando los cultivos se incubaron a temperaturas diferentes a 37 °C ^46^. No obstante, estas limitaciones se evitaron en esta investigación. Cabe anotar que el papel exacto de las hemolisinas en la infección por hongos no se ha dilucidado por completo ^40^.

Por otra parte, los resultados en la formación de biopelículas del presente estudio (82 %) fueron similares a los descritos por Pakshir *et al.*^15^, quienes encontraron biopelículas en el 62,7 % de los aislamientos; y la producción de biopelículas según su intensidad (débil, moderada fuerte), fue similar a la descrita por Mohammadi *et al.* en 2021 ^47^.

Las biopelículas han sido descritas como un factor importante de patogenicidad y resistencia en las levaduras ^48^^-^^49^ De hecho, algunos autores consideran que el crecimiento de hifas y la formación de biopelículas son los principales factores que optimizan la progresión de la patogenicidad en *Candida* spp. ^16^^,^^41^^,^^43^. Así, y a pesar de que la formación de biopelículas se asocia más comúnmente con *C. albicans,* este mecanismo también ha sido descrito en *C. glabrata, C. tropicalis, C. parapsilosis* y la levadura emergente multirresistente *C. auris*^50^^-^^53^.

A propósito de la producción de enzima coagulasa como factor de patogénesis, los resultados de esta investigación fueron similares a los descritos por Rodrigues *et al.*^24^, quienes no solo describieron la presencia de esta enzima en los aislamientos de *C. albicans* (88,5 %), sino también, en los de otras especies del género *Candida* (con excepción de *C. krusei),* que incluían aislamientos cepas de *C. tropicalis* (82,6 %) y *C. parapsilosis* (34,5 %), entre otras. De modo contrario, Seifi *et al.,* en 2015, no encontraron cepas productoras de coagulasa entre más de 100 aislamientos de diferentes especies de *Candida* (incluyendo 51 de *C. albicans),* aun con incubaciones de hasta 24 horas ^54^.

La producción de coagulasa se ha propuesto como una capa de protección frente a los macrófagos y se ha descrito en *C. parapsilosis, C. glabrata, C. guilliermondi,* entre otras especies del género ^24^^,^^55^.

Finalmente, en esta investigación todos los aislamientos fueron identificados como *C. albicans* mediante espectrometría de masas MALDI-TOF, lo que sugiere que la circulación y la frecuencia de aislamientos de las especies emergentes *C. dubliniensis* y *C. africana,* a partir de colecciones de cultivos o muestras vaginales, son infrecuentes.

Estos resultados coinciden con los obtenidos en 2012 en Nigeria, donde encontraron que solo dos de los 117 aislamientos en una colección de cultivos correspondían a *C. africana* y no encontraron aislamientos de *C. dubliniensis*^12^; o con los descritos por Theil *et al.,* en 2016 en Argentina, quienes determinaron la prevalencia de *Candida dubliniensis* (1,39 %) y *C. africana* (0.35 %) en una colección de 287 cepas ^1^. Asimismo, en 2017, Hazirolan *et al.* reexaminaron 376 aislamientos identificados como *C. albicans* y encontraron tres de *C. africana* y tres de *C. dubliniensis,* con una prevalencia de 0,8 % ^56^. Durante ese mismo año, Pakshir *et al.* realizaron un estudio para discriminar *C. africana* a partir de 110 cepas identificadas previamente como *C. albicans,* sin encontrar aislamientos de *C. africana*^15^.

Como se mencionó previamente, en el país, Rodríguez-Leguizamón *et al.* (2015) describieron una colección de aislamientos de *C. albicans* con variaciones atípicas; no obstante, la identificación molecular reveló correspondencia con *C. albicans* y los espectros de masas obtenidos por MALDI-TOF (utilizando el equipo MALDI Biotyper - Bruker Daltonics), no fueron concluyentes ^5^.

En conclusión, la presencia de *C. dubliniensis* y *C. africana* es escasa o nula en colecciones de cultivos o aislamientos previamente identificados como *C. albicans,* y la presencia de mecanismos de patogenicidad en dichos aislamientos es variable.

Debido a que ninguna prueba manual es completamente específica, las pruebas complementarias o comerciales son útiles para discriminar *C. albicans* y *C. dubliniensis.* En el caso de *C. africana,* y por ser una variedad de *C. albicans,* las pruebas moleculares son la herramienta necesaria para su identificación.
